# Integrated Metabolomic and Transcriptomic Analysis Reveals Tissue-Specific Secondary Metabolic Differentiation and Indole Alkaloid Accumulation in *Evodia rutaecarpa*

**DOI:** 10.3390/biology15171500

**Published:** 2026-09-02

**Authors:** Weiwei Zhao, Jihua Guo, Taihang Wang, Li Zhou, Guoyi Zhang, Yuanjiang Xu

**Affiliations:** 1 College of Biology and Chemistry, Minzu Normal University of Xingyi, Xingyi 562400, China; zhaoyuwei114@163.com (W.Z.);; 2 Guizhou Mountain Agricultural Machinery Research Institute, Guiyang 550000, China; 3 Chongqing Academy of Chinese Materia Medica, Nanshan Road, Huangjueya, Nanan District, Chongqing 400065, China

**Keywords:** *Evodia rutaecarpa*, widely targeted metabolomics, tissue specificity, indole alkaloids, UPLC-MS/MS, biosynthetic pathway

## Abstract

*Evodia rutaecarpa* is a valuable medicinal plant, yet its non-medicinal tissues remain largely underexplored. Here, we integrated ultra-performance liquid chromatography-tandem mass spectrometry (UPLC-MS/MS)-based widely targeted metabolomics and RNA sequencing (RNA-seq) transcriptomics to systematically profile the metabolic and transcriptional landscapes of roots, stems, leaves, and flowers of *Evodia rutaecarpa* (Juss.) Benth. Our aim was to characterize tissue-specific metabolic differentiation and its underlying transcriptional regulatory mechanisms. Metabolomic analysis, employing principal component analysis (PCA) and orthogonal partial least squares-discriminant analysis (OPLS-DA) with robust model parameters (R^2^Y > 0.9, Q^2^ > 0.5), identified 3090 differential metabolite features (variable importance in projection, VIP > 1.0; *p* < 0.05) across the four tissues, which exhibited distinct tissue-specific clustering patterns. Integrated Kyoto Encyclopedia of Genes and Genomes (KEGG) enrichment analysis and weighted gene co-expression network analysis (WGCNA) revealed that roots specifically accumulated quinolone alkaloids and flavonoid glycosides, accompanied by the coordinated upregulation of genes involved in flavonoid and phenylpropanoid biosynthetic pathways. In contrast, stems, leaves, and flowers were enriched in indole alkaloids (evodiamine and rutaecarpine) and volatile oil precursors, with concurrent upregulation of genes involved in tryptophan metabolism and indole alkaloid biosynthesis (e.g., tryptophan decarboxylase, *TDC*; s N-methyltransferase, NMT). Notably, leaves and flowers displayed particularly high accumulation levels of these bioactive alkaloids, suggesting their potential as alternative sources for industrial and pharmaceutical applications. WGCNA further identified multiple transcription factors and structural gene modules tightly correlated with evodiamine accumulation, offering promising candidate regulators for future biosynthetic pathway engineering. Collectively, this multi-omics integration study systematically elucidates the tissue-partitioned secondary metabolism of *Evodia rutaecarpa* (Juss.) Benth. and provides a solid scientific foundation for full-plant resource utilization, targeted development of non-medicinal tissues, and future metabolic engineering of indole alkaloid production.

## Simple Summary

*Evodia rutaecarpa* (Juss.) Benth. is a classic traditional Chinese medicinal plant with significant pharmacological activities, and its dried fruits are the officially recognized medicinal part. While most existing research focuses on the fruit, the metabolic and transcriptional characteristics of non-medicinal tissues (roots, stems, leaves, and flowers) remain underexplored, which hinders the full utilization of *E. rutaecarpa* plant resources. To address this gap, we conducted an integrated analysis combining widely targeted metabolomics and transcriptomics to profile the metabolic and transcriptional landscapes across four tissues of *E. rutaecarpa*. Our results revealed pronounced tissue-specific secondary metabolic differentiation: roots specifically accumulated quinolone alkaloids and flavonoid glycosides, whereas leaves, stems, and flowers preferentially accumulated bioactive indole alkaloids (evodiamine and rutaecarpine). Notably, leaves and flowers exhibited particularly high accumulation levels of these active alkaloids, indicating their potential as alternative sources for industrial and pharmaceutical production. We further identified key candidate structural genes and regulatory gene modules associated with indole alkaloid biosynthesis. Collectively, our findings provide a systematic foundation for the comprehensive utilization of *E. rutaecarpa* resources and lay a solid groundwork for future metabolic engineering of bioactive indole alkaloids.

## 1. Introduction

*Evodia rutaecarpa*, a perennial shrub or small tree belonging to the Rutaceae family and *Tetradium* genus, is primarily distributed in regions south of the Qinling Mountains in China. Its dried, nearly ripe fruit is the officially recognized medicinal part [1]. *Evodia rutaecarpa* boasts a long-standing medicinal history, with its Chinese name “Wu Zhu Yu” first recorded in the pre-Qin period text “Fifty-Two Diseases Prescriptions”. It is also documented in numerous classic pharmacological texts, including the Eastern Han Dynasty’s “Shen Nong’s Herbal Classic” [2]. According to the 2020 edition of the *Pharmacopoeia of the People’s Republic of China*, the traditional Chinese medicine “Wu Zhu Yu” derives from the dried near-mature fruits of *Evodia rutaecarpa* (Juss.) Benth., *E. rutaecarpa* var. *officinalis* (Dode) Huang, or *E. rutaecarpa* var. *Bodinieri* (Dode) Huang. This medicinal herb exhibits diverse therapeutic effects, including dispelling cold and relieving pain, suppressing adverse qi to stop vomiting, and assisting yang to stop diarrhea. It is clinically indicated for the treatment of jueyin headache, abdominal pain due to cold hernia, cold-dampness-induced foot edema, menstrual abdominal pain, epigastric distention and pain, vomiting with sour regurgitation, and early morning diarrhea [3].

*Evodia rutaecarpa* is widely utilized in clinical compound prescriptions. Notable classical formulas such as “Wu Zhu Yu Tang” (from *Treatise on Febrile Diseases*), “Zuo Jin Wan” (from *Danxi’s Heart Method*), “Wen Jing Tang” (from Jin Gui Yao Lue), “Dao Qi Tang” (from Yi Fang Jian Yi) and “Ji Ming San” (from *Principles of Diagnosis and Treatment*) all feature *Evodia rutaecarpa* as the monarch herb or an important minister herb, highlighting its significant position within the traditional Chinese medicine system [4]. Modern pharmacological studies have demonstrated that *Evodia rutaecarpa* exhibits a wide range of biological activities, encompassing anti-tumor, anti-inflammatory and analgesic, antioxidant, cardiovascular protection, gastrointestinal protection, neuroprotection, and hypoglycemic and hypolipidemic effects [5,6]. Among them, evodiamine (EVO) and rutaecarpine (RUT) are the most representative active constituents of *E. rutaecarpa*; both belong to indoloquinazoline alkaloids and exhibit significant anti-tumor activities [7,8]. EVO can induce tumor cell apoptosis and G1/S phase cell cycle arrest by activating the p53 signaling pathway, downregulating Bcl-2, and upregulating Bax expression [9]; furthermore, it can trigger G2/M phase cell cycle arrest through the JNK pathway [10]. These studies have validated the traditional efficacy of *E. rutaecarpa* at the molecular level, providing a scientific basis for its clinical application.

To date, more than 240 chemical constituents have been isolated and identified from *Evodia rutaecarpa*, mainly including alkaloids (ca. 133), terpenoids (ca. 36), steroids (ca. 5), phenolic compounds (ca. 51), and other structural types of compounds (ca. 15) [11]. The major active constituents of *Evodia rutaecarpa* are evodiamine and rutaecarpine. Accumulating evidence indicates that both compounds possess anti-cancer effects in vitro and in vivo [7].

The study of the biosynthetic pathways of active ingredients in *Evodia rutaecarpa* is an important foundation for understanding the formation mechanisms of its pharmacologically active substances, regulating the quality of the medicinal material, and achieving heterologous synthesis of active components. As early as the 1960s, Yamazaki et al. [12] confirmed through feeding experiments using isotopic labeling with carbon-14 that tryptophan, anthranilic acid, and formic acid are precursor substances for the biosynthesis of alkaloids in *Evodia rutaecarpa*. Subsequent research further elucidated that tryptophan undergoes a decarboxylation reaction to form tryptamine. Tryptamine then condenses with derivatives of anthranilic acid to form dihydronorharman, which subsequently reacts with anthranilic acid. Through a series of cyclization and oxidation modifications, it ultimately leads to the formation of evodiamine. Meanwhile, evodiamine can also be obtained by chemical synthesis, e.g., semisynthesis from rutaecarpine via N-methylation [13] or one-pot total synthesis through a continuous biscyclization reaction [14]. Although progress has been made in the chemical synthesis of active components of evodia and the tracking of biosynthetic precursors, the complete biosynthetic pathway has yet to be fully elucidated, and a considerable amount of research work remains to be carried out.

With the rapid advancement of multi-omics technologies, the integrated analysis of transcriptomics and metabolomics has emerged as a powerful approach for elucidating the regulatory mechanisms of secondary metabolism in medicinal plants [15,16]. These two omics technologies collectively reveal the causal relationship between gene expression changes and metabolic phenotypes from the perspectives of gene expression and metabolite accumulation, providing systematic evidence for deciphering complex metabolic regulatory networks [17]. Transcriptomics technology enables the qualitative and quantitative analysis of all transcripts in biological samples. In medicinal plant research, transcriptomic analysis can identify structural and regulatory genes involved in secondary metabolic pathways, revealing differential gene expression patterns across various tissues, developmental stages, or in response to environmental stresses [18]. Metabolomics technology allows for the comprehensive qualitative and quantitative analysis of small molecules in biological samples, directly reflecting the metabolic phenotype of an organism [19].

Despite its long history of application as a traditional Chinese medicine and its diverse pharmacological activities, current research on *Evodia rutaecarpa* (Juss.) Benth. still has some limitations. Existing studies primarily focus on the chemical components of the fruit, while research on the chemical constituents and metabolic characteristics of non-medicinal parts such as roots, stems, leaves, and flowers remains relatively scarce. Furthermore, there is a lack of research that systematically analyzes the relationship between gene expression and metabolite accumulation in *Evodia rutaecarpa* (Juss.) Benth. from a multi-omics integration perspective, making it difficult to comprehensively understand the overall landscape of secondary metabolic regulation [20].

Based on the aforementioned research background, this study focuses on *Evodia rutaecarpa* (Juss.) Benth. and employs a multi-omics strategy combining UPLC-MS/MS non-targeted metabolomics and RNA-seq transcriptomics to systematically compare and analyze the metabolite compositions and gene expression profiles of its roots, stems, leaves, and flowers. By integrating data from both omics approaches, this study aims to: (1) Elucidate the distribution patterns of metabolites in different tissues of *Evodia rutaecarpa* (Juss.) Benth., identify tissue-specific differential metabolites, and reveal the tissue-specific accumulation patterns of secondary metabolites; (2) Analyze the differences in gene expression among different tissues of *Evodia rutaecarpa* (Juss.) Benth. and identify key enzyme genes and regulatory genes involved in the biosynthetic pathways of major active components; (3) Construct a secondary metabolism regulatory network for *Evodia rutaecarpa* (Juss.) Benth. through gene-metabolite association analysis, particularly focusing on the biosynthetic pathways of indole quinolinone alkaloids. The findings of this study will contribute to a deeper understanding of the regulatory mechanisms underlying secondary metabolism in *Evodia rutaecarpa* (Juss.) Benth., providing theoretical support and genetic resources for the evaluation of medicinal material quality, comprehensive utilization of resources, and the biosynthesis of active ingredients. Additionally, this research will serve as a methodological reference for multi-omics studies on other medicinal plants.

## 2. Materials and Methods

### 2.1. Materials and Reagents

The samples of *Evodia rutaecarpa* used in this study were collected on 21 June 2025 from Wenjia Haizi, Zhengtun Town, Xingyi City, Guizhou Province, China (25°04′33″ N, 104°54′27″ E, elevation approximately 1190.5 m). This area is characterized by a subtropical monsoon climate, with an average annual temperature ranging from 16.5 to 18.0 °C and annual precipitation between 1200 and 1400 mm. Healthy individuals aged three years with consistent growth conditions were selected, and samples were taken from four different tissue types: mature leaves (Sa), flower buds (Sb), current year’s stems (Sc), and roots (Sd).

Immediately after collection, the samples were rinsed with sterile deionized water, and surface moisture was removed using absorbent paper. The samples were then rapidly frozen in liquid nitrogen for 10 min to inhibit metabolic activity. After freezing, the samples were transferred to a −80 °C ultra-low temperature freezer for storage until subsequent metabolomic and transcriptomic analyses.

Three biological replicates were set up for each tissue, and each replicate was a mixed sample from three different individual plants to reduce the influence of individual differences on metabolic and transcriptional results.

### 2.2. Widely Targeted Metabolomics Analysis

#### 2.2.1. Metabolite Extraction

After vacuum freeze-drying the tissue samples for 63 h, the samples were ground using a ball mill (MM 400, Retsch, Haan, Germany) at a frequency of 30 Hz for 90 s. A precise amount of 30 mg of the freeze-dried powder was weighed and mixed with 1.5 mL of pre-cooled 70% methanol aqueous solution (containing 250 μg/mL of internal standard). The mixture was vortexed and subjected to intermittent extraction in a low-temperature environment (vortexing for 30 s every 30 min, for a total of 6 cycles).

The extracted solution was then centrifuged at 12,000 rpm for 3 min, and the supernatant was filtered through a 0.22 μm micropore filter for further analysis. Quality control (QC) samples were prepared by mixing equal volumes of all samples to monitor system stability.

The reagents used in this study were of analytical grade: methanol (chromatography grade, Merck, Darmstadt, Germany), acetonitrile (chromatography grade, Merck, Darmstadt, Germany), formic acid (mass spectrometry grade, Sigma-Aldrich, St. Louis, MO, USA), and ultrapure water prepared using a Milli-Q system (Merck Millipore, Darmstadt, Germany).

#### 2.2.2. Chromatographic Separation Conditions

Chromatographic separation was performed on an Agilent 1290 Infinity LC system (Agilent Technologies, Santa Clara, CA, USA) equipped with an ACQUITY UPLC HSS T3 column (1.8 μm, 2.1 mm × 100 mm, Waters, Milford, MA, USA). The column temperature was maintained at 40 °C, and the flow rate was set to 0.4 mL/min. Mobile phase A consisted of 0.1% (*v*/*v*) formic acid in ultrapure water, and mobile phase B consisted of 0.1% (*v*/*v*) formic acid in acetonitrile. The gradient elution program was as follows: 0–2 min, 5–10% B; 2–15 min, 10–95% B; 15–17 min, 95% B; 17–17.1 min, 95–5% B; 17.1–20 min, 5% B. The injection volume was 2 μL for each sample.

Mass spectrometry was performed using an AB SCIEX Triple TOF 6600+ system (AB SCIEX, Framingham, MA, USA) equipped with an electrospray ionization (ESI) source operating in both positive and negative ionization modes. The ESI source parameters were set as follows: ion source gas 1 (GS1), 55 psi; ion source gas 2 (GS2), 55 psi; curtain gas (CUR), 35 psi; source temperature, 550 °C; ion spray voltage floating (ISVF), 5500 V in positive mode and −4500 V in negative mode; declustering potential (DP), 80 V; collision energy (CE), 10 eV and −10 eV for full scan acquisition.

#### 2.2.3. Metabolite Identification and Quantification

Based on a widely targeted metabolomics strategy, metabolite identification was performed using the MetWare Database (MWDB, Wuhan Maiwei Metabolic Biotechnology Co., Ltd., Wuhan, China), which contains manually curated spectral data for over 20,000 authentic reference standards. For each metabolite candidate, we performed multi-dimensional matching that included: (1) comparison of the measured accurate mass of the precursor ion with the theoretical mass in the database, with a mass tolerance of less than 5 ppm; (2) matching of the MS/MS fragmentation pattern between the detected metabolite and the database entry, requiring a matching score higher than 0.7; and (3) alignment of the experimental retention time with the database retention time (maximum deviation < 0.1 min) [21]. To ensure the accuracy of identification for the key bioactive indole alkaloids (evodiamine and rutaecarpine) that are the focus of this study, we further verified their identities using commercial authentic reference standards (purity > 98%, purchased from the National Institute for the Control of Pharmaceutical and Biological Products, Beijing, China) by comparing their retention times, precursor ions, and fragment ion patterns with those of the detected peaks.

For quantitative analysis, MultiQuant 3.0.2 software (AB SCIEX, Framingham, MA, USA) was employed for automated chromatographic peak integration of all metabolites detected in multiple reaction monitoring (MRM) mode. After integration, we performed manual inspection and correction for all peaks to ensure accurate integration boundaries, particularly for low-abundance metabolites with overlapping peaks. Inter-sample retention time correction was performed based on the internal standards that were added uniformly to all samples during extraction, which adjusted for minor retention time shifts introduced by column aging and solvent system variation across batches. The relative content of each metabolite was quantified using the peak area of the corresponding MRM transition, and all peak areas were normalized to the peak area of the internal standard (250 μg/mL lidocaine) to correct for any systematic variation introduced during sample preparation and instrumental analysis [22]. To ensure data reliability, metabolites that were not detected in more than 50% of the samples or had a coefficient of variation (CV) greater than 50% across all quality control (QC) samples were excluded from further statistical analysis.

#### 2.2.4. Data Preprocessing and Statistical Analysis

Raw data underwent missing value imputation, where missing values were replaced with 1/5 of the minimum detected value for each metabolite. Metabolites with a coefficient of variation (CV) ≥ 0.5 in quality control (QC) samples were subsequently excluded. The retained metabolites were then subjected to unit variance scaling (UV scaling).

Multivariate statistical analysis was performed using R software (v4.1.2). Principal component analysis (PCA) was conducted using the prcomp function, extracting principal components based on UV scaling to examine the overall distribution trend of samples and intra-group dispersion [23,24]. Orthogonal partial least squares discriminant analysis (OPLS-DA) was performed using the MetaboAnalystR package [25]. Data were log_2_-transformed and mean-centered, and model validity was assessed through a permutation test with 200 iterations to prevent overfitting [26].

The criteria for selecting differential metabolites were: variable importance in projection (VIP) > 1.0, and fold change (FC) ≥ 2 or ≤0.5.

These threshold criteria were selected based on the conventional standards established in widely targeted metabolomics research to ensure that we identified differentially accumulated metabolites with both statistical robustness and biological significance. A VIP value greater than 1.0 indicates that the metabolite contributes more to the inter-group separation than the average metabolite in the OPLS-DA model, which helps prioritize metabolites that are primarily responsible for the observed metabolic differentiation among different tissues. The fold change cutoff of ≥2 or ≤0.5 was chosen to focus on metabolites that exhibit at least a two-fold difference in accumulation between tissue groups, which effectively filters out minor variations that are likely due to technical noise and retains only those metabolites with substantial, biologically meaningful changes in abundance that are more likely to be associated with tissue-specific functional differences.

#### 2.2.5. Metabolic Pathway Enrichment Analysis

Differential metabolites were mapped to the KEGG database [27] and the MetMap pathway database. The significance of metabolic pathway enrichment was assessed using hypergeometric tests. The degree of enrichment was measured by the Rich Factor and *p*-value, enabling analysis of metabolic network reprogramming characteristics across different tissues.

This section explains why hypergeometric distribution tests were used to assess the significance of metabolic pathway enrichment. The hypergeometric test is a standard statistical approach for evaluating whether the number of differential metabolites annotated to a specific pathway is significantly greater than would be expected by chance, given the total number of metabolites detected in the study and the total number of metabolites in that pathway. This method is well-suited for pathway enrichment analysis because it accounts for the varying sizes of different metabolic pathways, which helps avoid false positive enrichment results caused by large pathways containing more metabolites overall. By using this test, we can robustly identify pathways that are truly over-represented among the differential metabolites, providing statistically reliable insights into the metabolic network reprogramming underlying tissue-specific differentiation.

### 2.3. Transcriptomics Analysis

#### 2.3.1. RNA Extraction and Library Construction

Total RNA from various tissues was extracted using the Trizol method. After quality assessment via agarose gel electrophoresis, Qubit 4.0 fluorometry, and Qsep400 bioanalyzer (Guangding Biotech, Taiwan, China), mRNA was enriched using oligo (dT) magnetic beads. The cDNA library was constructed through a series of steps including fragmentation, double-stranded cDNA synthesis, end repair, adenine tailing, adaptor ligation, PCR amplification, and denaturation-circularization. The effective concentration of the library was accurately quantified (>2 nM) after quantification with Qubit and verification of insert fragment size with an Agilent 2100 (Agilent Technologies, Santa Clara, CA, USA).

#### 2.3.2. Sequencing and Data Quality Control

Following DNB preparation, the library was loaded onto the MGI high-throughput sequencing platform and subjected to paired-end sequencing using the PE150 strategy. Raw sequencing data were filtered using fastp (v0.23.2) to remove adapter sequences [28], reads containing more than 10% N bases, and low-quality reads (where Q ≤ 20 bases accounted for >50%), resulting in clean reads for subsequent analysis.

#### 2.3.3. Sequence Assembly and Functional Annotation

Clean reads were de novo assembled using Trinity (v2.15.1) with a k-mer length of 25 [29]. Transcript clustering and redundancy removal were performed using Corset (v1.09) to obtain a Unigene sequence set [30]. Unigenes were aligned with databases including NR, Swiss-Prot, TrEMBL, KEGG, KOG, and GO using DIAMOND (v2.0.9) [31]. Additionally, protein domain annotations were performed using HMMER with the Pfam database. The completeness of the assembly was evaluated using BUSCO (v4.5.5).

#### 2.3.4. Gene Expression Quantification and Differential Analysis

Clean reads were mapped to the Unigene reference sequences using RSEM (v1.3.1) in combination with Bowtie2 (v2.4.5) to calculate the FPKM (Fragments Per Kilobase of transcript per Million mapped reads) values for each gene [32,33]. Differential expression analysis between groups was performed using DESeq2 (v1.22.2) [34], with the screening thresholds set as |log_2_Fold Change| ≥ 1 and FDR (False Discovery Rate) < 0.05. Subsequently, the differentially expressed genes were subjected to GO (Gene Ontology) functional classification [35], KEGG (Kyoto Encyclopedia of Genes and Genomes) pathway enrichment analysis [36], and KOG (euKaryotic Orthologous Groups) clustering analysis [37].

#### 2.3.5. Weighted Gene Co-Expression Network Analysis (WGCNA)

WGCNA was performed to construct a gene co-expression network. The optimal soft threshold was selected using the pickSoftThreshold function to ensure a scale-free network fit index (R^2^) > 0.85. Hierarchical clustering based on the Topological Overlap Matrix (TOM) identified gene modules using dynamic tree cutting (merge cut height = 0.25, minimum module gene number = 50). Module eigengenes were calculated and correlated with tissue phenotypes to identify tissue-specific modules and hub genes.

Based on the gene functional annotation results, we systematically identified transcription factor (TF) families encoded in the *Evodia rutaecarpa* transcriptome. Hub TFs within tissue-specific co-expression modules were further extracted, which are potential key regulators controlling the expression of structural genes involved in secondary metabolite biosynthesis. These TFs, such as members of the bHLH, WRKY, and MYB families, provide candidate targets for future functional validation and metabolic engineering. Additionally, we performed simple sequence repeat (SSR) identification from the assembled unigene sequences using MISA software (https://webblast.ipk-gatersleben.de/misa/, accessed on 12 August 2026). A total of numerous SSR loci were detected, which can be developed into molecular markers for subsequent genetic diversity assessment, germplasm identification, and marker-assisted breeding studies in *Evodia rutaecarpa*. These genomic resources lay an important foundation for future genetic improvement and conservation research of this important medicinal plant species.

#### 2.3.6. Integrative Analysis of Transcriptomics and Metabolomics

An integrative analysis of differentially expressed genes and metabolites was performed based on the KEGG database to construct a gene-metabolite co-regulatory network. Pearson correlation analysis was conducted to identify genes significantly associated with key metabolites (|r| > 0.8, *p* < 0.05). This analysis, combined with WGCNA module membership, aimed to uncover key enzyme genes and transcription factors regulating the accumulation of tissue-specific metabolites.

## 3. Results and Analysis

### 3.1. Results of Widely Targeted Metabolomics Analysis

**Figure 1:**
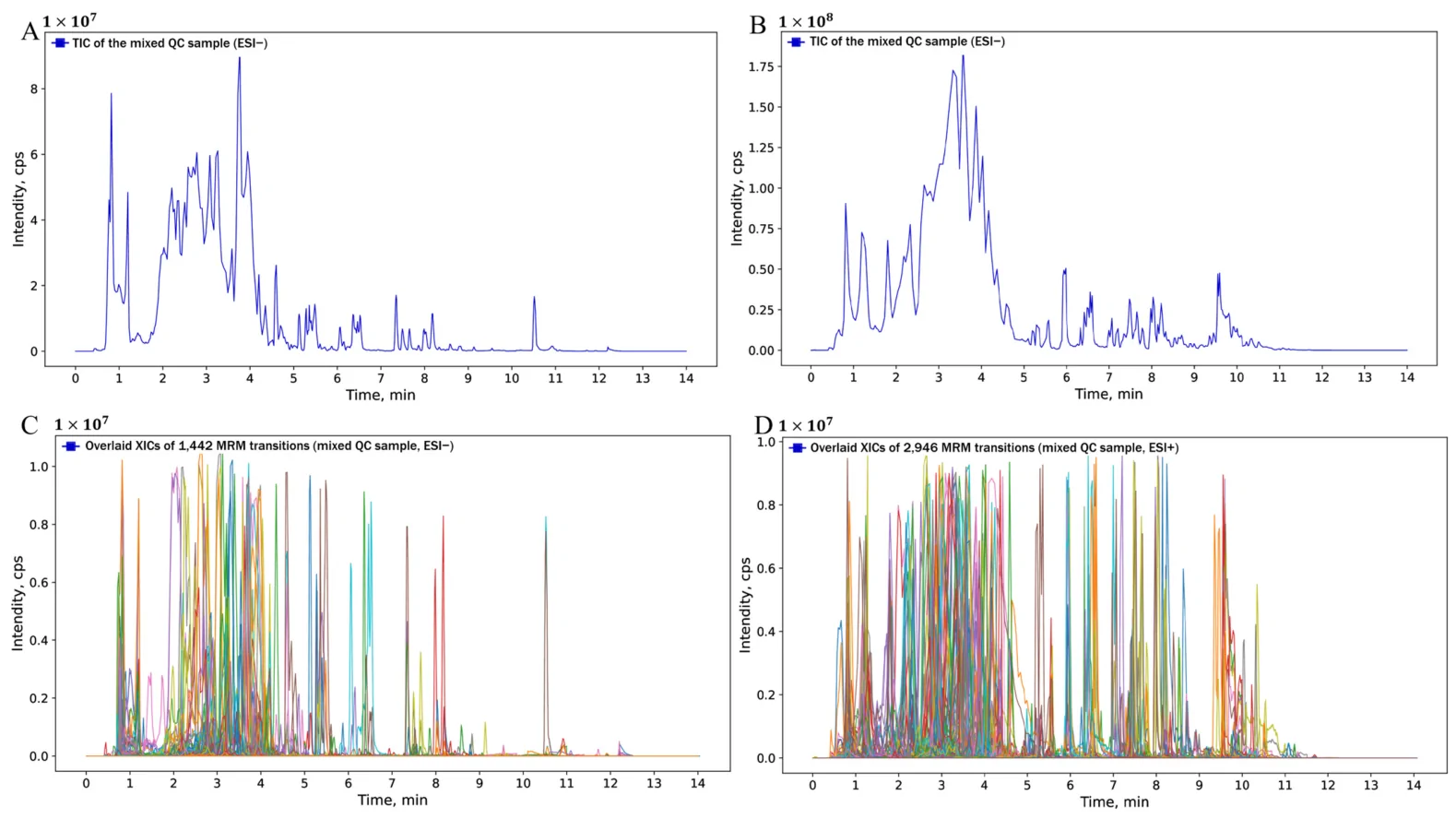
Representative total ion chromatogram (TIC) and MRM extracted ion chromatograms (MRM-XIC) from the widely targeted metabolomics analysis of *Evodia rutaecarpa*. (**A**) Total ion chromatogram (TIC) of the mixed QC sample in negative ion mode (1,442 MRM transitions); (**B**) TIC of the mixed QC sample in positive ion mode (2,946 MRM transitions); (**C**) Overlaid extracted ion chromatograms (XICs) of all MRM transitions in negative ion mode, where each colored trace represents one metabolite ion pair; (**D**) Overlaid XICs of all MRM transitions in positive ion mode. Note: In (**C**,**D**), each chromatographic peak of a different color represents a detected metabolite.

**Figure 2:**
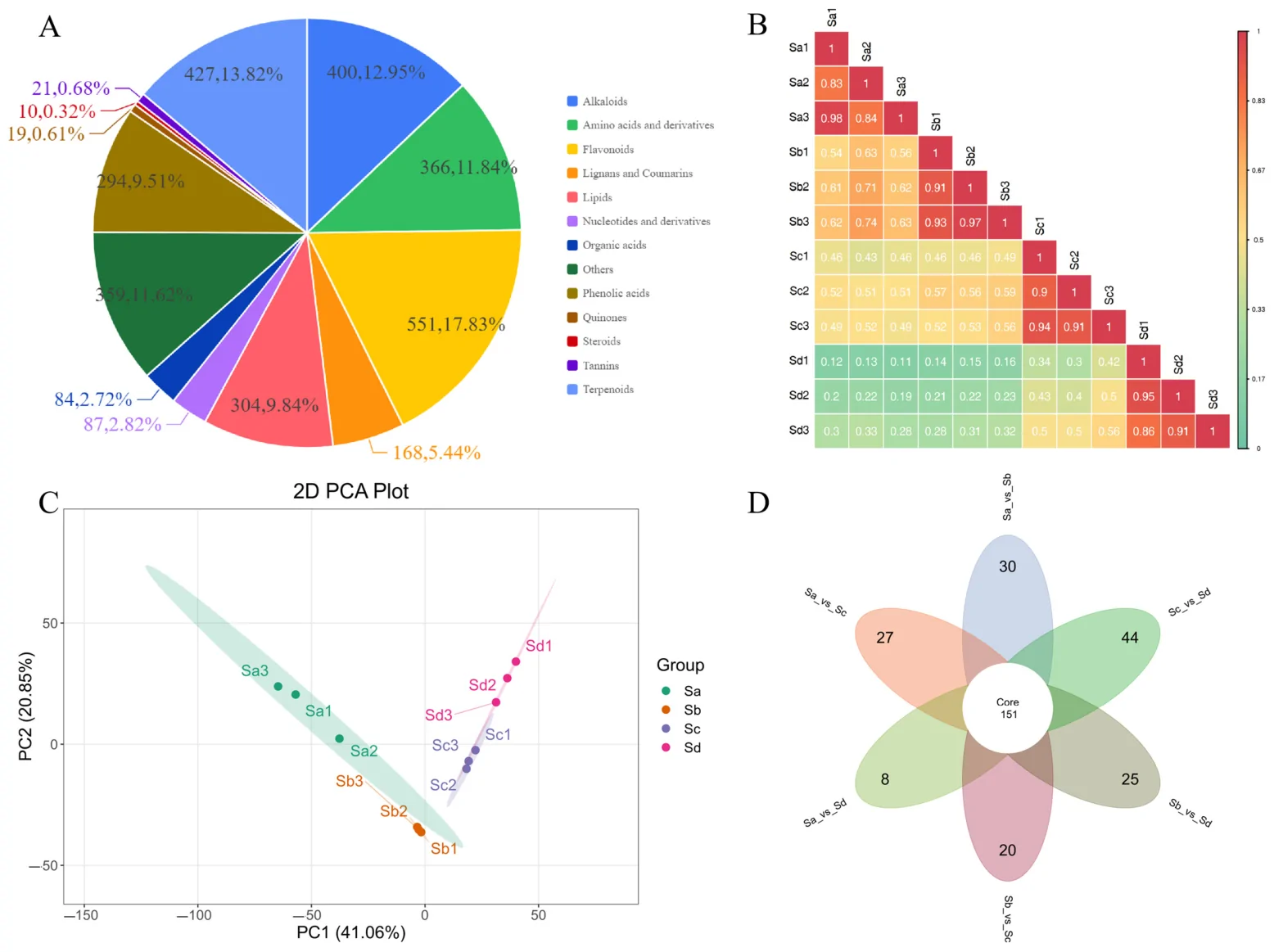
Metabolome analysis of *E. rutaecarpa* (**A**) Pie chart of metabolite classification, (**B**) Pearson’s correlation coefficient heatmap, (**C**) Principal component analysis score plot, and (**D**) Venn diagram showing the number of shared and tissue-specific metabolites of *E. rutaecarpa*.

Using a UPLC-MS/MS detection platform to perform widely targeted metabolomics profiling, we observed high-quality overlap in the total ion chromatograms (TIC) of quality control (QC) samples for the *Evodia rutaecarpa* metabolome. This result confirms that the instrument stability and data reproducibility met the requirements for subsequent downstream analysis (Figure 1). In total, we detected 3090 metabolites across four tissues of *E. rutaecarpa* (leaves, flowers, stems, and roots), which were classified into 13 major metabolite classes including alkaloids, flavonoids, terpenoids, amino acids and their derivatives, lipids, and phenolic acids (Figure 2A).

#### 3.1.1. Widely Targeted Metabolomics Profiling of *Evodia rutaecarpa*

In this study, we performed widely targeted metabolomics analysis on four distinct tissues of *Evodia rutaecarpa*: root, stem, leaf, and flower. In total, 3090 metabolites were identified and classified into 13 major functional classes. Consistent with the typical metabolic profile of medicinal plants, secondary metabolites dominated the overall composition, with the most abundant classes being flavonoids (551 metabolites, 17.83%), terpenoids (427, 13.82%), alkaloids (400, 12.95%), phenolic acids (294, 9.51%), and lignans and coumarins (168, 5.44%). Primary metabolites were primarily represented by amino acids and their derivatives (366, 11.84%), lipids (304, 9.84%), glycosides and their derivatives (87, 2.82%), and organic acids (84, 2.72%) (Figure 2A).

We next assessed sample correlation using Pearson’s correlation coefficient (r). The results revealed that correlation coefficients between biological replicates within each tissue were all higher than 0.84 (Sa: 0.84–0.98; Sb: 0.91–0.97; Sc: 0.90–0.94; Sd: 0.86–0.95), confirming good intra-group repeatability and reliable data quality. In contrast, the correlation coefficients between root (Sd) and the other three tissues ranged only from 0.11 to 0.56, indicating strong tissue-specific metabolic signatures (Figure 2B).

Principal component analysis (PCA) was then performed to visualize global metabolic variation. The first two principal components (PC1 and PC2) explained 41.06% and 35.52% of the total metabolic variance, respectively, yielding a cumulative contribution rate of 76.58%. Notably, PC1 clearly separated the samples into two distinct clusters: one cluster containing root and stem (Sc and Sd), and the other containing leaf and flower (Sa and Sb). The PC2 axis further resolved metabolic differentiation between roots and stems, as well as between flowers and leaves. The PCA results were consistent with the conclusions from the Pearson correlation analysis, collectively demonstrating that *Evodia rutaecarpa* exhibits significant metabolic differentiation across different tissues (Figure 2C).

To further characterize the metabolic differentiation among roots, stems, leaves, and flowers, we analyzed differential metabolites using a Venn (petal) diagram. The results showed that 151 core differential metabolites were shared across all pairwise comparisons. Among the comparison-specific differential metabolites, stem versus root (Sc vs. Sd) exhibited the highest number of unique metabolites (44), followed by leaf versus flower (Sa vs. Sb, 30), leaf versus stem (Sa vs. Sc, 27), flower versus root (Sb vs. Sd, 25), flower versus stem (Sb vs. Sc, 20), and leaf versus root (Sa vs. Sd, 8) (Figure 2D).

#### 3.1.2. Hierarchical Clustering Analysis of Tissue-Specific Metabolic Profiles

**Figure 3:**
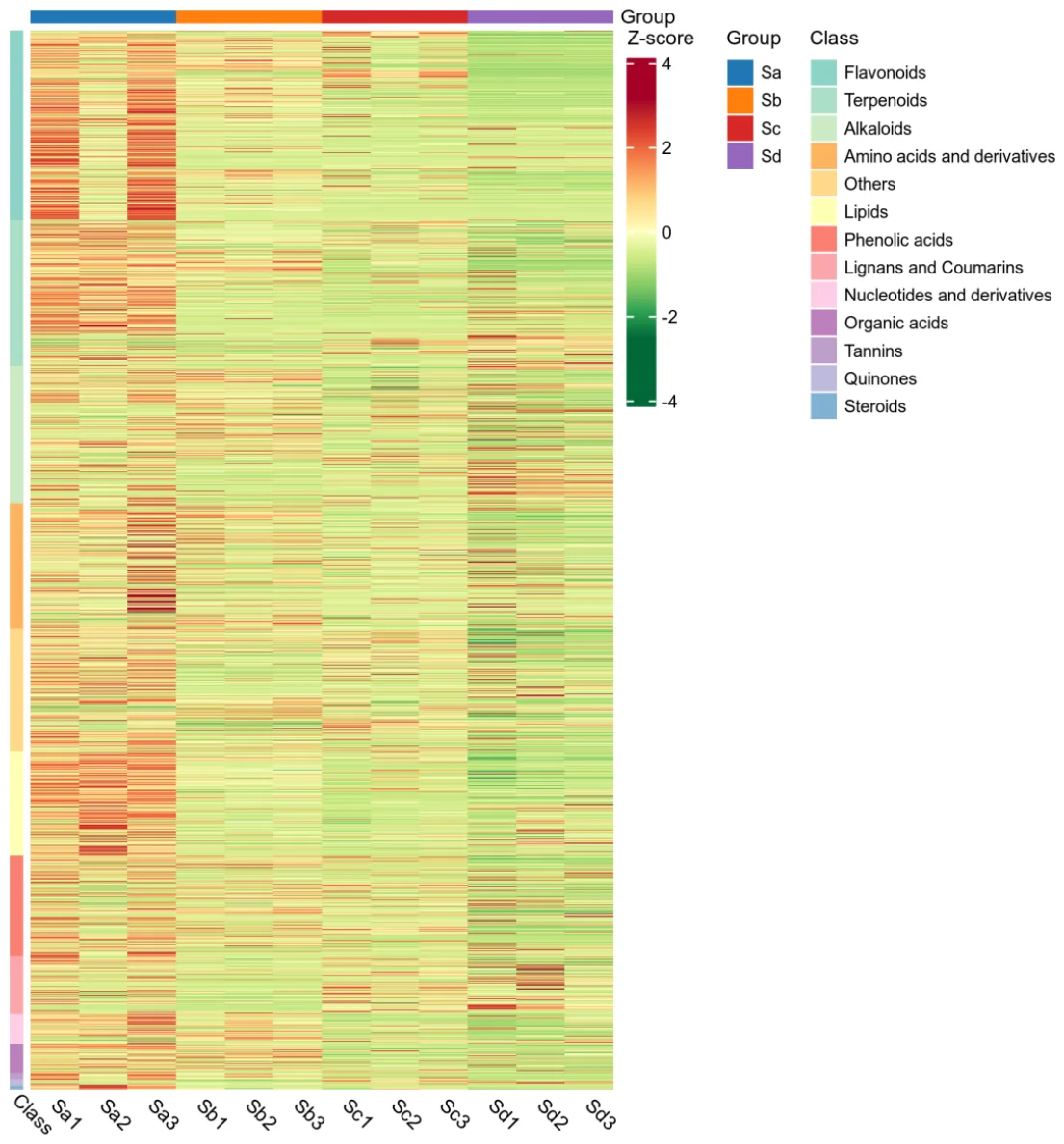
Cluster heatmap of metabolites across different tissues of *E. rutaecarpa.* Note: Horizontal axis represents sample names, vertical axis represents metabolite information, and groups are indicated by clustering. Colors correspond to Z-score standardized relative contents, with red indicating high accumulation and green indicating low accumulation.

To further characterize the tissue-specific metabolic patterns across the four tissues of *Evodia rutaecarpa*, we performed hierarchical clustering analysis (HCA) on the 3090 identified metabolites using Z-score standardized data and generated a heatmap to visualize global metabolic divergence (Figure 3). The clustering results confirmed high reproducibility within each biological replicate group, with leaves (Sa), flowers (Sb), stems (Sc), and roots (Sd), forming four distinct, well-separated metabolic clusters, clearly demonstrating the existence of strong tissue-specific metabolic differentiation. Root tissues exhibited a broad-spectrum metabolic profile characterized by high accumulation of multiple metabolite classes: the deep red coloration in the heatmap indicated significantly elevated levels of alkaloids, amino acids, lipids, and phenolic acids, which is consistent with the well-documented role of plant roots as primary storage organs for specialized metabolites. Stem tissues showed partial metabolic similarity to roots but displayed a substantially reduced accumulation of alkaloids compared to root tissues. Leaf tissues exhibited generally lower overall metabolic intensity, with only specific flavonoid glycosides showing high accumulation (marked by deep red coloration). This pattern aligns with the core function of leaves as photosynthetic source organs, which prioritize primary metabolism for growth and typically export synthesized secondary metabolites to sink tissues. Floral tissues displayed a unique metabolic signature: most metabolites showed low background accumulation, but terpenoids (specifically limonoids) and alkaloids (indole quinazolines) exhibited specific, strong upregulation, as visualized by distinct deep red bands in the heatmap. This tissue-specific distribution pattern suggests that floral organs preferentially allocate metabolic resources to defensive secondary metabolites to protect reproductive tissues and ensure successful reproductive development.

#### 3.1.3. Analysis of Differential Metabolites

**Figure 4:**
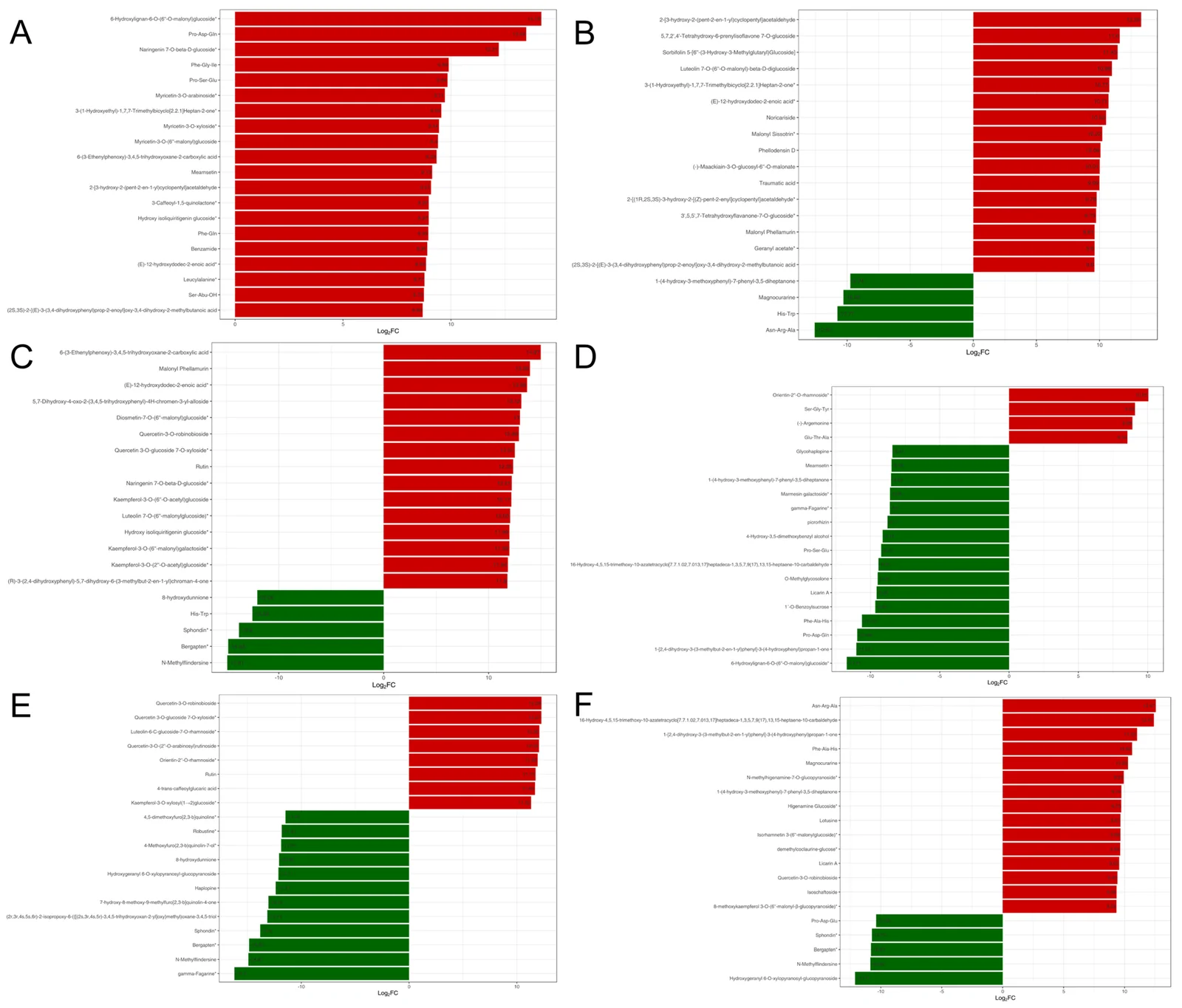
TopFc Bar plots showing differential metabolite accumulation in pairwise tissue comparisons of *E. rutaecarpa* (**A**) Sa vs. Sb; (**B**) Sa vs. Sc; (**C**) Sa vs. Sd; (**D**) Sb vs. Sc; (**E**) Sb vs. Sd; (**F**) Sc vs. Sd. * Isobaric isomers that could not be distinguished by the applied UPLC-MS/MS method.

To identify tissue-specific metabolic markers, we conducted differential metabolite analysis using the orthogonal partial least squares discriminant analysis (OPLS-DA) model, with the following stringent significance thresholds: variable importance in projection (VIP) > 1, absolute log_2_ fold change (|log_2_FC|) ≥ 1, and *p*-value < 0.05 (Figure 4). Pairwise cross-tissue comparisons revealed that the most pronounced metabolic divergence occurred between leaves and roots (Sa vs. Sd). Leaves were significantly enriched in flavonoid glycosides (e.g., quercetin-3-O-robinobioside, kaempferol malonyl glycoside, rutin) and isoquinoline alkaloids, with log_2_FC values of some differentially accumulated metabolites reaching as high as 14.97. In contrast, roots showed significantly higher accumulation of only a small set of specialized metabolites, including bergapten, sphondin, N-methylflindersine, and hydroxygeraniol diglycoside. We further observed a striking difference in metabolic profiles between (Sa vs. Sd): leaves are highly enriched in flavonoids, including Quercetin-3-O-robinobioside (log_2_FC = 12.89), Quercetin 3-O-glucoside 7-O-xyloside, and Rutin. Roots, on the other hand, are rich in coumarins (Bergapten, log_2_FC–14.83; Sphondin, log_2_FC–13.8) and alkaloids (N-Methylflindersine, 8-hydroxydunnione, and His-Trp).

#### 3.1.4. Tryptophan Metabolism and Indole Alkaloid Biosynthesis

Tryptophan is a pivotal aromatic amino acid in plants, serving as a core output molecule of the shikimic acid pathway with functionally divergent metabolic fates [38]. In primary metabolism, tryptophan is converted to auxin (IAA) via the indole-3-pyruvic acid (IPA) pathway to regulate plant growth and development [39]. In secondary metabolism, tryptophan undergoes decarboxylation and oxidation to enter the indole alkaloid biosynthesis pathway, acting as the skeleton donor for indole quinazoline alkaloids (e.g., evodiamine, rutaecarpine) and terpenoid indole alkaloids (e.g., camptothecin) [40]. In this study, we identified 400 alkaloid metabolites across roots, stems, leaves, and flowers of.

**Figure 5:**
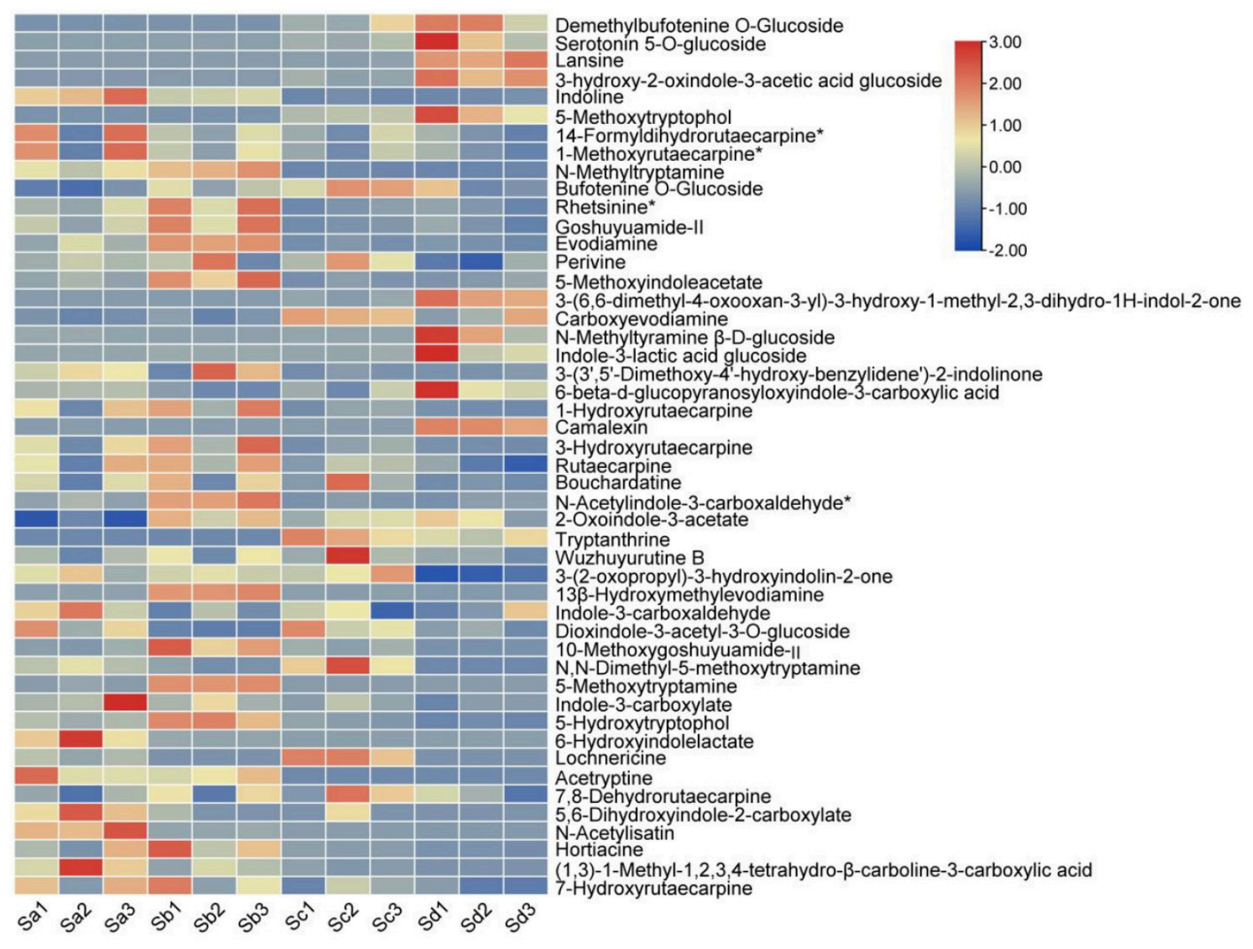
Tissue-specific accumulation patterns of indole alkaloids in *E. rutaecarpa*. * Isobaric isomers that could not be distinguished by the applied UPLC-MS/MS method; values represent the summed signal of all isomers.

*E. rutaecarpa*, representing a significant portion of the total metabolome. Indole alkaloid profiles exhibited striking tissue specificity (Figure 5). Hierarchical cluster analysis revealed that root and stem metabolic profiles were highly similar, while leaves and flowers formed a distinct separate cluster. Quantitative analysis based on mass spectrometry peak areas showed that flower tissues accumulated the highest levels of evodiamine-type alkaloids, including evodiamine, 13β-hydroxymethylevodiamine, and rhetsinine.

Notably, 13β-hydroxymethylevodiamine had a tissue specificity index of 0.93, indicating that flowers are the primary accumulation organ for these bioactive components. Indole precursors such as indoline and N-acetylisatin were highly enriched in leaves, suggesting a key role for leaf tissues in synthesizing the indole alkaloid skeleton. Glycosylated indole derivatives (e.g., serotonin 5-O-glucoside, demethylbufotenine O-glucoside) were specifically enriched in root tissues, with tissue specificity indexes all exceeding 0.70, indicating that glycosylation modification is preferentially activated in roots. Collectively, these results demonstrate functional differentiation in indole alkaloid synthesis and accumulation across root, leaf, and flower tissues, highlighting leaves and flowers as critical medicinal tissues for indole alkaloid production, complementary to the traditionally used fruits.

### 3.2. Transcriptome Analysis Results

#### 3.2.1. Transcriptome Profile of *Evodia rutaecarpa*

**Figure 6:**
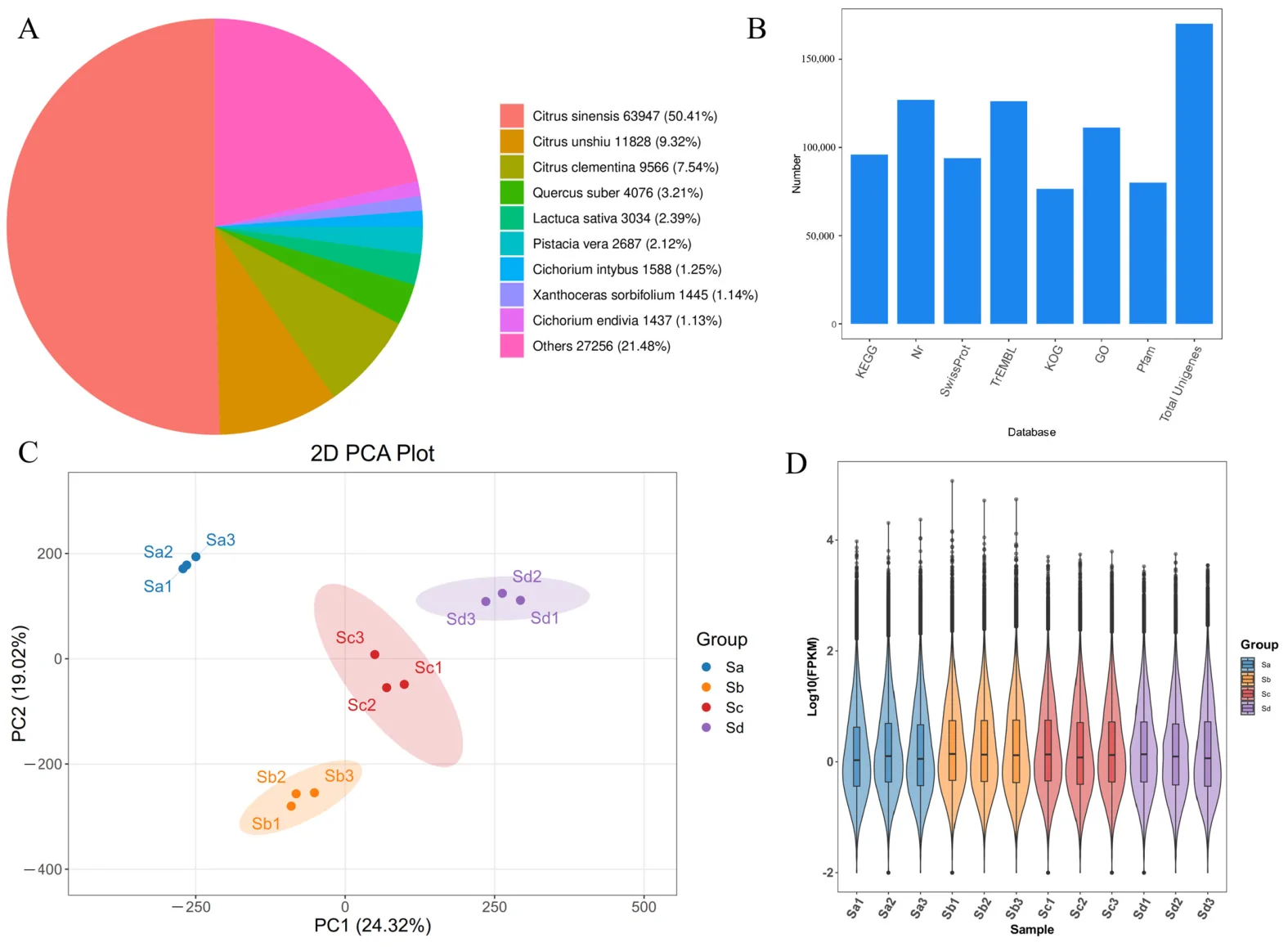
Transcriptome characterization of *E. rutaecarpa* tissues (**A**) Species annotation distribution pie chart; (**B**) Functional annotation statistics bar chart; (**C**) Principal component analysis score plot; (**D**) Violin plot of FPKM expression levels.

To characterize the transcriptional landscape of *Evodia rutaecarpa*, we constructed 12 RNA sequencing libraries representing four tissue types (roots, stems, leaves, flowers) with three biological replicates each. After quality filtering, the number of clean reads per library ranged from 45,054,308 to 81,135,600, with an average of 61,826,946.67 reads per sample. The average GC content was 43.63%, and the average Q20 and Q30 rates were 99.26% and 97.36%, respectively, indicating high sequencing quality (Table 1). De novo transcriptome assembly using these high-quality reads generated a comprehensive reference transcriptome with an average transcript length of 1315 bp and an N50 length of 1907 bp, yielding a total of 170,135 unigenes. Species annotation analysis based on the NR database showed that the majority of *E. rutaecarpa* unigenes aligned to Rutaceae species, particularly citrus species, with *C. Sinensis* (50.41%), *C. unshiu* (9.32%), and *C. Clementina* (7.54%) accounting for 67.27% of total annotations, consistent with the close phylogenetic relationship between Evodia and Citrus genera. Notably, 21.48% of unigenes showed no significant homology to known sequences, suggesting the presence of numerous Rutaceae-specific or Evodia-specific genes (Figure 6A). Integrated annotation across KEGG, Nr, SwissProt, TrEMBL, KOG, GO, and Pfam databases revealed that 129,583 (76.16%) unigenes were annotated in at least one database (Figure 6B). The Nr and TrEMBL databases had the highest annotation rates (74.57% and 74.15%, respectively), indicating that most unigenes have known protein homologs. GO, Pfam, and KOG databases annotated 65.42%, 46.99%, and 44.98% of unigenes, respectively, providing a solid foundation for subsequent gene function classification, domain analysis, and orthologous gene family studies. Principal component analysis (PCA) of transcriptome data showed that the first two principal components (PC1 = 24.32%, PC2 = 19.02%) explained 43.34% of the total transcriptome variation, with clear intra-cluster clustering of biological replicates (Figure 6C). On the PC1 axis, leaves (Sa) and roots (Sd) were positioned at opposite ends, showing the greatest transcriptional divergence, reflecting significant differences in global gene expression patterns between vegetative and reproductive organs. flowers (Sb) and Stems (Sc) were distributed in the intermediate region. The consistent expression patterns and good reproducibility among samples confirmed the reliability of the transcriptome data for subsequent differential expression analysis. A small number of highly expressed genes were identified in flowers (Sb) samples, potentially associated with vigorous secondary metabolic processes such as evodiamine biosynthesis (Figure 6D).

#### 3.2.2. KOG and GO Functional Annotation Analysis

To systematically classify gene functions, we performed KOG and GO annotation analyses. Based on the KOG database, a total of 2640 unigenes were annotated to the “Secondary metabolite biosynthesis, transport, and catabolism” category (Class Q), directly forming a structural gene library for the synthesis of bioactive components in *E. rutaecarpa*, including indole quinazoline alkaloids, limonoids, and flavonoid glycosides. Genes involved in amino acid metabolism (Class E, 3318 unigenes) and lipid metabolism (Class I, 3148 unigenes) provide essential precursors for alkaloid and terpenoid biosynthesis, respectively. Carbohydrate metabolism genes (Class G, 4345 unigenes) support extensive glycosylation modifications of secondary metabolites. Transcription-related genes (Class K, 4769 unigenes) and signal transduction genes (Class T, 4504 unigenes) constitute a complex metabolic regulatory network. GO annotation further confirmed at the molecular function level that genes associated with catalytic activity and transporter activity each numbered approximately 50,000, providing a functional basis for the enzymatic reactions and compartmental storage of secondary metabolites. Notably, a high proportion of unigenes were annotated as “Function unknown” (Class S, 8582 unigenes) and “General function prediction only” (Class R, 14,416 unigenes), suggesting that *E. rutaecarpa* harbors numerous novel synthase genes unique to Rutaceae, which require further investigation through integrated metabolomics and WGCNA module analysis.

**Figure 7:**
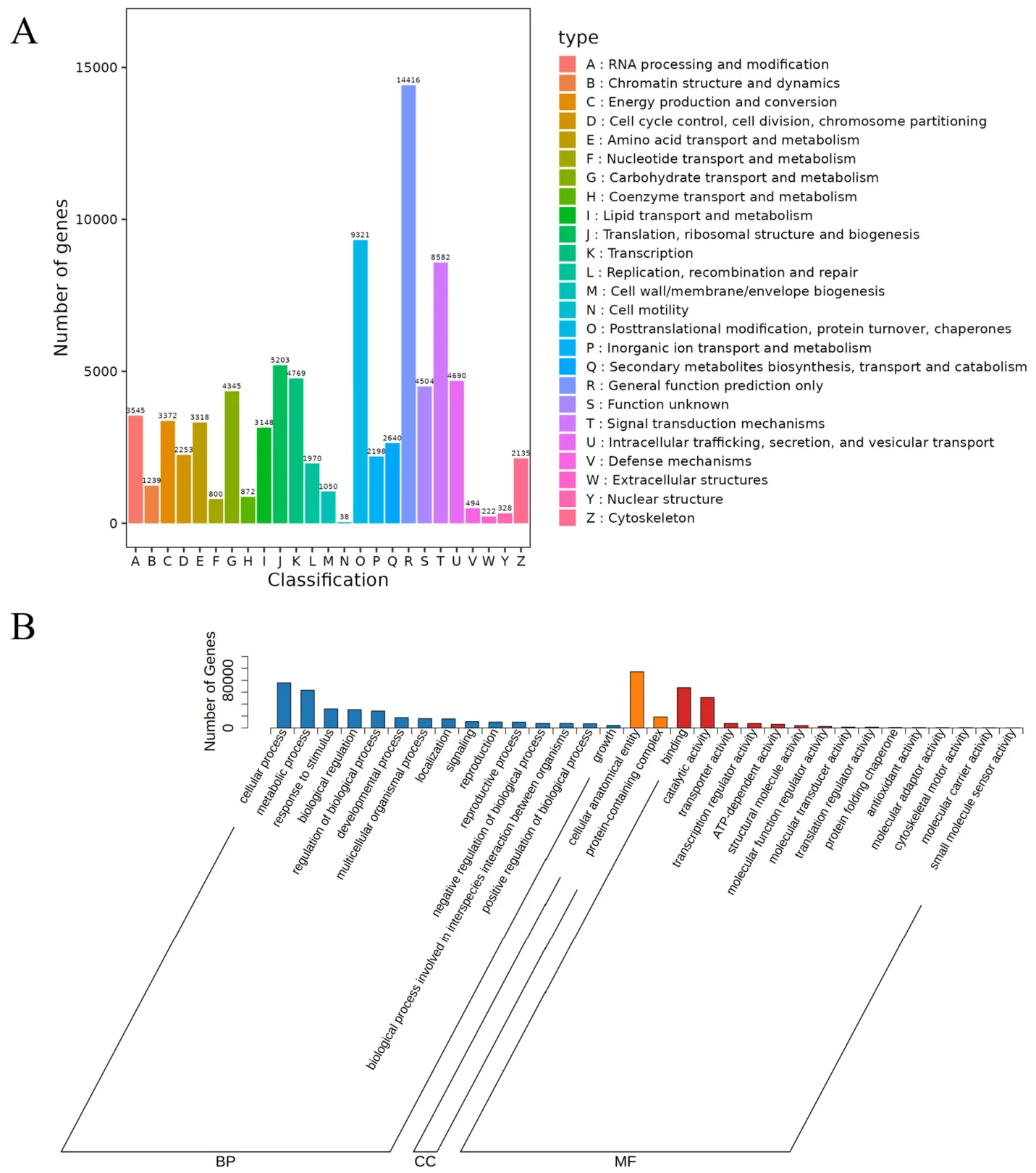
Functional classification of *E. rutaecarpa* unigenes. (**A**) KOG functional classification of unigenes; (**B**) GO functional classification of unigenes.

KOG functional classification results showed that 76,532 unigenes were distributed across 26 functional categories (Figure 7A). Among these, the “General function prediction only” (Class R) and “Function unknown” (Class S) categories accounted for the highest proportion, together representing 30.0% of annotated unigenes, suggesting that *E. rutaecarpa* contains a large number of species-specific or novel genes. Categories with abundant gene representation included “Posttranslational modification, protein turnover, chaperones” (Class O), “Signal transduction mechanisms” (Class T), and “Transcription” (Class K), providing rich resources for investigating tissue-specific regulatory networks. GO functional annotation results (Figure 7B) showed that in the biological process (BP) category, cellular processes and metabolic processes were the most highly enriched. In the molecular function (MF) category, binding and catalytic activity were dominant, indicating that a large number of genes in the *E. rutaecarpa* transcriptome are involved in enzymatic reactions and regulatory factor binding, consistent with its complex secondary metabolic characteristics.

#### 3.2.3. Integrated Metabolome and Transcriptome Analysis of *E. rutaecarpa*

**Figure 8:**
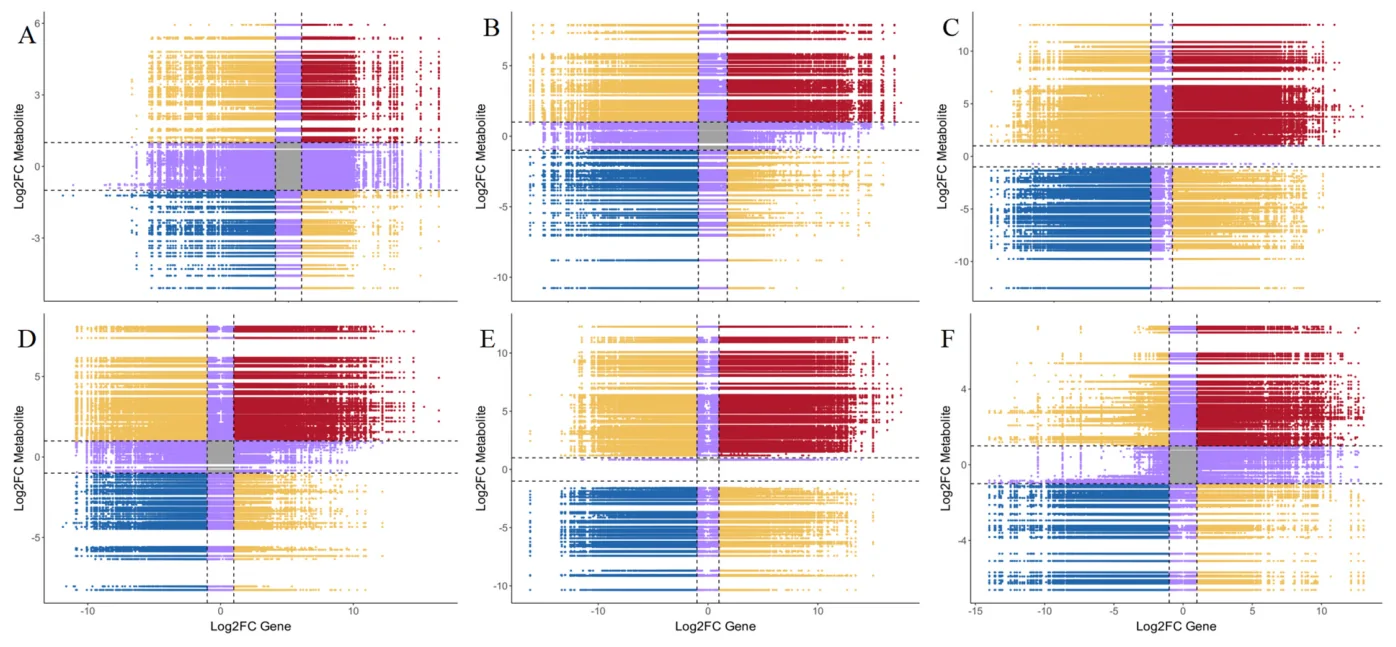
Nine-quadrant association plots of differentially expressed genes (DEGs) and differentially accumulated metabolites (DAMs) across six pairwise tissue comparisons in *E. rutaecarpa* (**A**) Leaf (Sa) versus flower (Sb), (**B**) leaf (Sa) versus stem (Sc), (**C**) leaf (Sa) versus root (Sd), (**D**) flower (Sb) versus stem (Sc), (**E**) flower (Sb) versus root (Sd), (**F**) stem (Sc) versus root (Sd) X-axis indicates Log_2_ fold change (FC) of genes; Y-axis indicates Log_2_ FC of metabolites. Gray dots in the central sector (where both |log_2_FC| values are below the threshold) represent genes and metabolites without significant differences; purple dots represent DAMs with unchanged genes (top and bottom middle sectors) or DEGs with unchanged metabolites (left and right middle sectors); red dots represent positively correlated DEGs and DAMs (upregulated genes and upregulated metabolites); dark blue dots represent positively correlated DEGs and DAMs (downregulated genes and downregulated metabolites); yellow dots represent negatively correlated DEGs and DAMs. The Pearson correlation coefficient between transcriptomic and metabolomic profiles was >0.8 in all nine quadrant plots.

To integrate transcriptomic and metabolomic data, we filtered genes and metabolites based on Pearson correlation coefficient (PCC) values > 0.8, performed log_2_ transformation on the gene expression and metabolite accumulation data, and constructed nine-quadrant plots to visualize the associations between differentially expressed genes and differentially accumulated metabolites for each pairwise tissue comparison (Figure 8). In these plots, the first quadrant (genes upregulated/metabolites upregulated, represented by red dots) and the third quadrant (genes downregulated/metabolites downregulated, represented by blue dots) showed dense clustering of data points. This pattern indicates that the accumulation of most differential metabolites is positively correlated with the expression changes of structural genes in their corresponding biosynthetic pathways. In pairwise comparisons involving floral organs (Sb versus Sd), metabolites exhibited the largest magnitude of accumulation change, with log_2_FC values reaching up to ±10 (Figure 8E). This observation aligns with the specific enrichment of glycosylated metabolites in roots, suggesting that specific glycosyltransferase (UGT) genes are preferentially activated in roots tissues to drive this metabolite modification and accumulation. In contrast, the second and fourth quadrants (represented by yellow dots) contained relatively few data points, indicating that negative regulatory interactions between gene expression and metabolite accumulation are not the dominant factor driving inter-tissue metabolic divergence in *Evodia rutaecarpa*.

#### 3.2.4. Weighted Gene Co-Expression Network Analysis (WGCNA)

**Figure 9:**
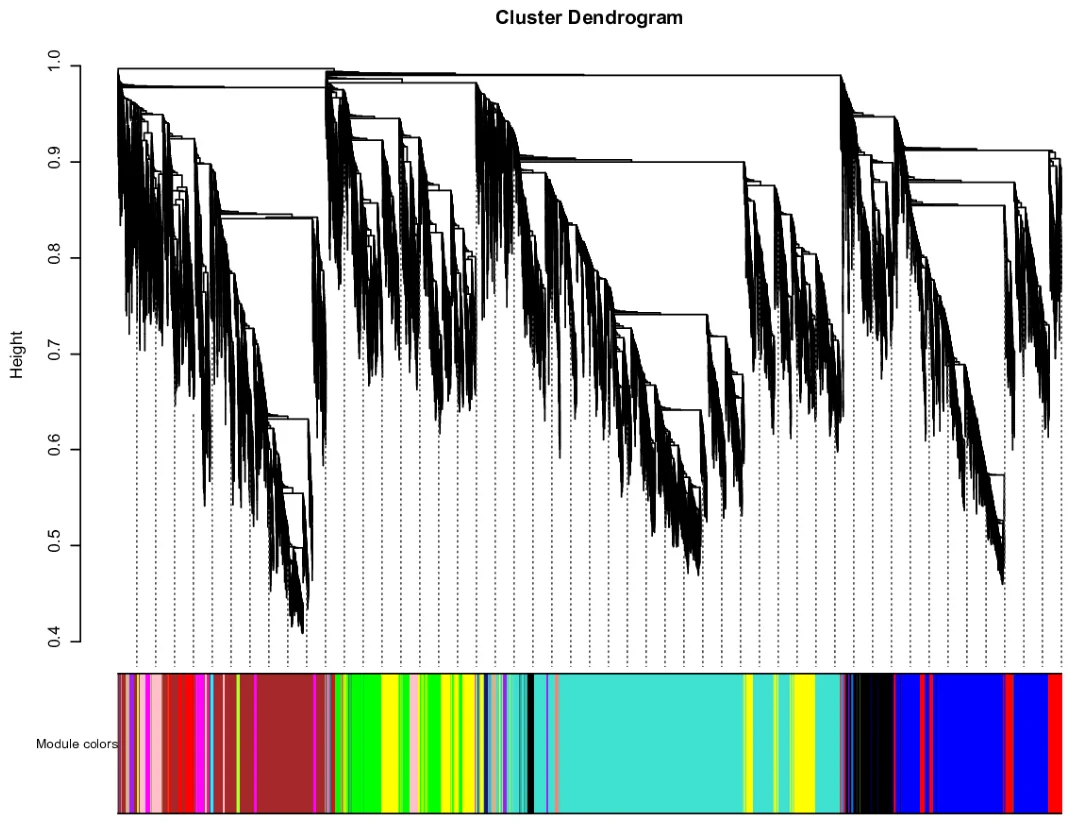
WGCNA co-expression network analysis of *E. rutaecarpa transcriptome.* The WGCNA analysis plot shows the gene dendrogram (**top**) and module clustering results (**bottom**), with each color denoting a specific module.

To identify tissue-specific gene regulatory networks, we performed weighted gene co-expression network analysis (WGCNA) using transcriptome data from root, stem, leaf, and flower tissues of *E. rutaecarpa* (Figure 9). Hierarchical clustering analysis revealed that the 12 samples clustered strictly according to tissue type, with biological replicates within each tissue showing high consistency, indicating reliable data quality suitable for subsequent co-expression network construction. Following soft-thresholding selection (R^2^ > 0.85), dynamic tree cutting, and module merging (merge cut height = 0.25), a total of 15 gene co-expression modules were identified, designated as black, blue, brown, cyan, green, greenyellow, magenta, midnightblue, pink, purple, red, salmon, tan, turquoise, and yellow; genes that were not clustered into any module were assigned to the grey module.

Module–trait correlation analysis revealed that several modules exhibited significant tissue-specific expression patterns. The core gene of the purple module was annotated as a MATE family multidrug efflux transporter (Cluster-20223.9). This family is widely implicated in the vacuolar compartmentalization and transmembrane transport of alkaloids in plants, suggesting that this module may mediate the directed accumulation and storage of evodiamine and rutaecarpine in specific tissues. The core gene of the greenyellow module was annotated as an NAC domain-containing transcription factor (Cluster-52506.2). As a core regulator of plant secondary metabolism, its expression pattern was significantly associated with root tissue, suggesting that it may participate in the biosynthetic regulation of evodiamine and rutaecarpine by activating the expression of structural genes in the indoloquinazoline alkaloid biosynthetic pathway.

### 3.3. Biosynthesis Pathway Analysis of Evodiamine

**Figure 10:**
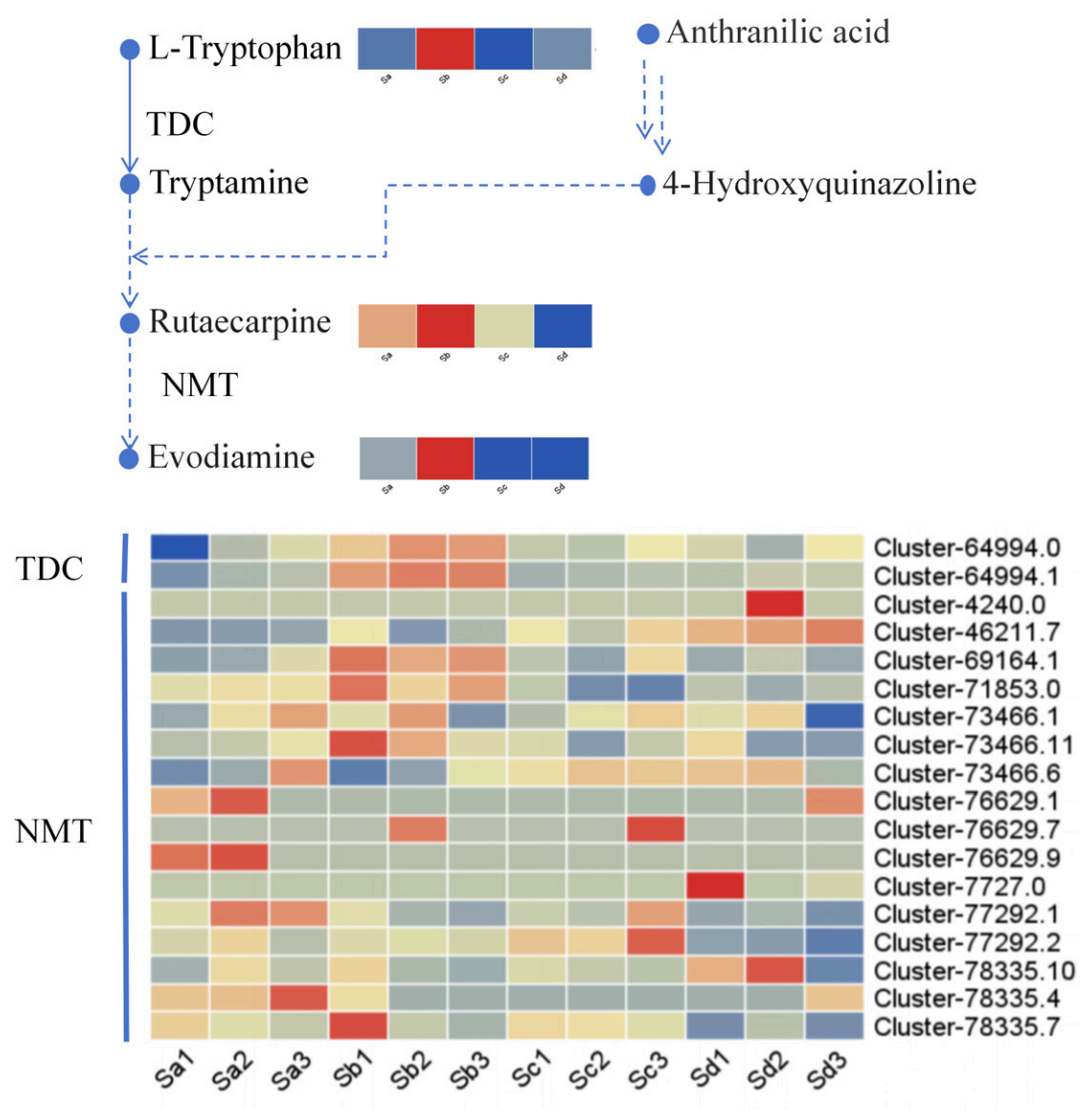
Proposed biosynthesis pathway of evodiamine and rutaecarpine in *E. rutaecarpa.* Abbreviations: TDC, tryptophan decarboxylase; NMT, N-methyltransferase. Solid arrows indicate resolved biosynthetic steps with experimentally characterized enzymes; dashed arrows indicate unresolved biosynthetic steps for which the intermediate enzymes remain uncharacterized.

The biosynthetic pathways of evodiamine and rutaecarpine, two major bioactive indole alkaloids in *Evodia rutaecarpa*, were first elucidated through classical isotope feeding and chemical degradation studies conducted by Yamazaki and Ikuta [12]. These early studies established that tryptophan and anthranilic acid act as common precursors for alkaloid biosynthesis, with a one-carbon unit (C1-unit) carried by the tetrahydrofolate (THF) cycle participating in the construction of the 3-position of the indoloquinazoline core skeleton. Subsequently, an S-adenosylmethionine (SAM)-dependent N-methyltransferase (NMT) catalyzes the N-14 methylation of rutaecarpine to yield evodiamine. Building on this foundational work, our integrated transcriptomic and metabolomic analysis confirms that evodiamine biosynthesis follows a shikimate pathway-derived double-branch assembly and post-modification strategy (Figure 10). In the tryptophan branch, tryptophan is first decarboxylated by tryptophan decarboxylase (TDC) to produce tryptamine, which is then converted to indole-3-acetaldehyde via an amine oxidase-mediated cascade reaction to generate the indole moiety of the final alkaloid. In the parallel anthranilic acid branch, anthranilic acid undergoes cyclization with the THF-carried C1-unit to form the 4(3H)-quinazolinone moiety. Critically, the detection of tryptophan, anthranilate glycosides, S-adenosylmethionine, and a series of intermediate indole derivatives in our metabolome analysis provides direct in vivo metabolic evidence supporting this proposed biosynthetic pathway. At the transcriptome level, the coordinated high expression of TDC and NMT genes specifically in flower tissues, together with the specific activation of this pathway in the WGCNA turquoise module, confirms the molecular basis underlying why flowers function as the primary site for evodiamine synthesis and accumulation. As illustrated in Figure 10, the heatmaps embedded in the pathway topology reveal distinct tissue-specific accumulation patterns for the indoloquinazoline alkaloids. Notably, the color intensity for evodiamine, rutaecarpine, and 13β-hydroxymethylevodiamine is markedly stronger in flower tissues (Sb) compared to leaf (Sa), stem (Sc), and root (Sd) samples, suggesting that flowers represent the primary accumulation site for this alkaloid class. Consistent with this metabolic pattern, the transcriptome-level heatmaps demonstrate that multiple members of the TDC and NMT gene families exhibit coordinated high expression specifically in flower tissues, with the corresponding WGCNA turquoise module showing specific activation in this organ. This precise transcriptome–metabolome correspondence establishes the molecular basis underlying why flowers function as the primary site for evodiamine synthesis and accumulation.

## 4. Discussion

In this study, we employed an integrated multi-omics approach combining widely targeted metabolomics and transcriptomics to systematically profile the metabolic and transcriptional landscapes of four different tissues (roots, stems, leaves, and flowers) of *Evodia rutaecarpa*. Our results revealed significant tissue-specific metabolic differentiation and provided new insights into the spatial distribution of secondary metabolites, particularly the accumulation patterns of pharmaceutically important indole alkaloids. These findings expand our understanding of secondary metabolism in this traditional Chinese medicinal plant and have important implications for its sustainable utilization.

Our results clearly demonstrated that *Evodia rutaecarpa* exhibits distinct tissue-specific metabolic differentiation, with roots preferentially accumulating flquinolone alkaloids and coumarins, while leaves and flowers, are predominantly enriched in indole alkaloids (evodiamine and rutaecarpine). This tissue-specific partitioning of secondary metabolism is consistent with the general developmental and ecological principles of plant secondary metabolism, where different organs often specialize in the production and accumulation of specific classes of metabolites to fulfill distinct biological functions. In a previous study on the metabolome of *Evodia rutaecarpa* fruits during different developmental stages, Du et al. [41] reported dynamic changes in the accumulation of active components during fruit maturation, but the distribution patterns across different vegetative and reproductive tissues remained poorly characterized. Our study fills this knowledge gap by providing a comprehensive comparison of metabolite accumulation across four major tissues, revealing that flowers accumulate the highest levels of evodiamine and rutaecarpine, followed by leaves, while roots and stems contain markedly lower amounts of these indoloquinazoline alkaloids.

This finding has important practical implications for the utilization of *Evodia rutaecarpa* resources. Traditionally, only the near-mature fruits are used as the medicinal part in Chinese medicine, and the stems, leaves, and other tissues are often discarded during harvesting and processing. Our results clearly demonstrate that leaves contain substantial amounts of evodiamine and rutaecarpine, which are the primary bioactive components responsible for the pharmacological effects of *Evodia rutaecarpa*. Given that leaves are an annually renewable resource with much greater biomass than fruits, they can serve as a sustainable alternative source for the industrial extraction of these indole alkaloids. This not only increases the economic value of *Evodia rutaecarpa* cultivation but also helps reduce the pressure on wild populations and contributes to the conservation of this medicinal plant species. Similar findings have been reported in other medicinal plants, where non-medicinal tissues were found to contain high levels of bioactive compounds, highlighting the importance of full-plant utilization.

From a biosynthetic perspective, our integrative analysis of transcriptomic and metabolomic data confirmed the classical biosynthetic pathway of evodiamine and rutaecarpine that was proposed based on early isotope labeling studies. We found that genes encoding key enzymes in the indole alkaloid biosynthetic pathway, such as tryptophan decarboxylase (TDC) and strictosidine synthase (STR), were coordinately upregulated in leaves and flowers, which is consistent with the high accumulation of indole alkaloids observed in these tissues. This positive correlation between gene expression and metabolite accumulation provides in vivo evidence supporting the proposed biosynthetic pathway and identifies candidate structural genes that can be used for future metabolic engineering efforts to improve indole alkaloid production. In recent years, with the rapid development of synthetic biology, there has been increasing interest in the heterologous production of plant natural products in microbial hosts [42]. Our transcriptome data provides a rich source of gene candidates for reconstructing the evodiamine biosynthetic pathway in heterologous systems.

Weighted gene co-expression network analysis (WGCNA) identified several gene modules that are significantly associated with the accumulation of indole alkaloids, including a purple module with a core gene annotated as a MATE family transporter and a green-yellow module with an NAC transcription factor. MATE transporters have been shown to play important roles in the vacuolar sequestration and transport of alkaloids in many plant species [43], suggesting that this transporter may be involved in the directed accumulation and storage of evodiamine and rutaecarpine in specific tissues. NAC transcription factors are well-known regulators of secondary metabolism in plants, and recent studies have shown that they can activate the expression of structural genes in alkaloid biosynthetic pathways [44]. The identification of these candidate regulators provides a starting point for future functional studies to elucidate the transcriptional regulatory mechanisms that control tissue-specific indole alkaloid accumulation in *Evodia rutaecarpa*.

Despite these significant findings, our study also has some limitations that should be acknowledged. First, this is a cross-sectional study based on single-time-point sampling during the flowering stage, which does not capture the dynamic changes in metabolite accumulation that may occur during different growth and developmental stages or in response to different environmental conditions. It is well known that the content of secondary metabolites in medicinal plants is strongly influenced by seasonal variations, environmental factors, and developmental stage [45]. Future studies should therefore incorporate multi-time-point sampling across different seasons and growth stages to provide a more comprehensive understanding of the metabolic dynamics in *Evodia rutaecarpa*. Second, while we have identified candidate genes and regulatory modules associated with indole alkaloid biosynthesis based on correlation analysis, the functional roles of these genes remain to be validated through experimental approaches such as gene silencing, overexpression, and in vitro enzyme assays. Biochemical characterization of key enzymes and transcription factors will be essential to fully elucidate the molecular mechanisms that control the biosynthesis and accumulation of indole alkaloids in this plant. Third, the relative quantification approach used in this study provides information on the relative differences in metabolite accumulation across tissues, but absolute quantification using authenticated reference standards would provide more accurate quantitative data that can be directly used for quality control and standardization. Future work should therefore include absolute quantification of key bioactive components across different tissues and different origins to validate our findings.

In conclusion, our integrated transcriptomic and metabolomic analysis provides a comprehensive landscape of tissue-specific secondary metabolism in *Evodia rutaecarpa*. The results clearly demonstrate that non-medicinal tissues, particularly leaves, contain high levels of pharmaceutically active indole alkaloids, supporting the development of these tissues as alternative sources for the production of these valuable bioactive compounds. The candidate genes and regulatory modules identified in this study provide a rich resource for future functional genomics studies and metabolic engineering efforts aimed at improving indole alkaloid production. Overall, these findings contribute to the more rational and sustainable utilization of *Evodia rutaecarpa* resources and lay a solid foundation for future research on the biosynthesis and regulation of indole alkaloids in this important medicinal plant.

## 5. Conclusions

Based on RNA next-generation sequencing and UPLC-MS/MS-based metabolomics, this study systematically characterized the transcriptomic and metabolomic landscapes of four tissues (roots, stems, leaves, flowers) of *Evodia rutaecarpa* (Juss.) Benth. (the currently accepted name is Tetradium ruticarpum (A. Juss.) T.G. Hartley). Our integrative multi-omics analysis yielded the following major conclusions:

Tissue-specific metabolic divergence defines the secondary metabolite architecture of *Evodia rutaecarpa* (Juss.) Benth. Unsupervised and supervised multivariate analyses collectively revealed that the four tissues possess markedly distinct metabolomic profiles, with clear tissue-specific clustering patterns. Notably, roots and stems exhibited relatively closer metabolic similarity, as did leaves and flowers—a topological pattern that likely reflects the combined effects of developmental proximity (roots/stems as below- vs. above-ground vegetative organs, leaves/flowers as reproductive/assimilatory aerial tissues) and differential expression of tissue-enriched biosynthetic gene clusters. This spatial compartmentalization of metabolism underscores the sophisticated regulatory mechanisms governing secondary metabolite allocation in this medicinal plant species.

Differential accumulation of pharmaceutically relevant alkaloids enables resource reallocation. Indole alkaloids—particularly the pharmacologically active pair evodiamine and rutaecarpine—were found to be highly enriched in leaves and flowers. This finding carries dual significance: first, it challenges the conventional reliance on fruits as the sole source of these bioactive alkaloids; second, it provides compelling metabolomic evidence supporting the feasibility of utilizing leaves—an annually renewable and substantially more abundant non-medicinal tissue—as an alternative, sustainable source for the industrial extraction of evodiamine and rutaecarpine. Given that fruit harvesting often conflicts with plant regeneration and involves labor-intensive collection, leaf-based sourcing may offer a more ecologically and economically viable strategy.

Integrated pathway analysis bridges transcriptional regulation to metabolic output. KEGG enrichment analysis of differentially expressed genes (DEGs) coupled with differentially accumulated metabolites (DAMs) delineated a tissue-partitioned pathway activation landscape: Flavonoid and phenylpropanoid biosynthesis pathways were predominantly upregulated in roots, consistent with the leaves and flowers accumulation of flavonoid glycosides—a finding that aligns with the well-established role of roots as primary sites for phenylpropanoid-derived defense compound storage in many Rutaceae species. Tryptophan metabolism and indole alkaloid biosynthesis pathways were preferentially enriched in stems, leaves, and flowers, corresponding precisely to the elevated accumulation of evodiamine and rutaecarpine in these aerial tissues. This coordinated transcript-metabolite elevation suggests that the biosynthetic capacity for indole alkaloids is not restricted to fruits but is distributed across multiple above-ground organs, with leaves potentially serving as an underappreciated biosynthetic reservoir. Cutin, suberine, and wax biosynthesis pathways were specifically enhanced in flowers, likely associated with their terpenoid-rich epicuticular wax profiles and consistent with the ecological role of floral surfaces in pollinator attraction and desiccation resistance. Collectively, these pathway-level correlations provide a mechanistic framework for understanding how transcriptional reprogramming drives tissue-specific metabolic specialization in *Evodia rutaecarpa* (Juss.) Benth.

Broader implications for sustainable utilization and quality control. Beyond the immediate findings, this study has several translational ramifications: Full-plant valorization. By systematically mapping the metabolic repertoire of all four major organs, we provide a comprehensive chemical blueprint that facilitates the rational exploitation of currently discarded or underused tissues (stems, leaves, and even roots), thereby supporting a zero-waste paradigm for *Evodia rutaecarpa* (Juss.) Benth. cultivation and processing. Tissue-specific quality control. The marked metabolic heterogeneity observed across tissues underscores the inadequacy of current quality control standards that treat the plant as a uniform biological entity. Future pharmacopoeial specifications should consider tissue-specific reference markers and allowable ranges, particularly if non-fruit tissues are to be incorporated into medicinal or nutraceutical supply chains. Biotechnological perspectives. The tissue-enriched biosynthetic pathways identified here offer candidate targets for synthetic biology or metabolic engineering approaches aimed at enhancing the production of high-value indole alkaloids in heterologous or cultivated systems. Limitations and future directions. While this study provides a comprehensive multi-omics snapshot of tissue-level metabolic differentiation, several questions remain open for future investigation. First, the regulatory nodes (e.g., transcription factors, non-coding RNAs) governing the tissue-specific activation of indole alkaloid biosynthesis warrant deeper functional validation via targeted gene silencing or overexpression experiments. Second, the developmental dynamics of these tissue-specific metabolic profiles across different phenological stages—from seedling to fruiting—remain to be characterized, as do the environmental modulators (e.g., light, temperature, soil microbiota) that may influence secondary metabolite partitioning. Third, targeted absolute quantification of evodiamine and rutaecarpine across all tissues—ideally using authenticated reference standards and multiple batches of plant material from distinct geographical origins—will be essential to validate the quantitative comparisons reported here.

In conclusion, this integrative transcriptomic and metabolomic investigation systematically elucidates the tissue-partitioned secondary metabolism of *Evodia rutaecarpa* (Juss.) Benth., revealing that non-medicinal aerial parts—particularly leaves—harbor substantial accumulations of pharmaceutically active indole alkaloids at levels comparable to those found in conventional fruit-based materials. These findings collectively provide a multi-dimensional scientific foundation for the full-plant resource utilization of *E. rutaecarpa*, the sustainable and targeted development of its non-medicinal tissues, and the future establishment of tissue-specific quality control criteria that better reflect the intrinsic chemical heterogeneity of this valuable medicinal plant. This study serves as a preliminary multi-omics exploration of tissue-specific metabolites in *E. rutaecarpa*. Further biochemical verification of key transcription factors and gene functions will be conducted in future work to clarify the regulatory mechanism of indole alkaloid biosynthesis.

## Figures and Tables

**Table 1:** Summary of sequencing statistics of *E. rutaecarpa*.

Sample	Raw Reads	Raw Base (G)	Clean Reads	Clean Base (G)	Q20 (%)	Q30 (%)	GC Content (%)
Sa1	71,983,088	10.8	71,014,032	10.65	99.32	97.58	43.36
Sa2	53,334,084	8	53,136,836	7.97	99.2	97.14	43.46
Sa3	56,940,440	8.54	56,075,076	8.41	99.36	97.69	43.63
Sb1	55,408,542	8.31	54,582,528	8.19	99.28	97.43	43.81
Sb2	57,362,278	8.6	55,922,478	8.39	99.29	97.45	43.98
Sb3	57,230,814	8.58	55,527,762	8.33	99.34	97.64	43.94
Sc1	64,667,442	9.7	63,624,382	9.54	99.19	97.13	43.17
Sc2	83,612,720	12.54	81,135,600	12.17	99.26	97.36	43.88
Sc3	66,441,644	9.97	64,960,662	9.74	99.3	97.51	43.37
Sd1	69,523,102	10.43	68,343,286	10.25	99.09	96.81	43.77
Sd2	74,205,808	11.13	72,546,410	10.88	99.31	97.52	43.33
Sd3	46,552,110	6.98	45,054,308	6.76	99.19	97.15	43.86
average	63,105,172	9.46	61,826,946	9.27	99.26	97.36	43.63

## Data Availability

The raw transcriptome sequencing data generated in this study have been deposited in the NCBI Sequence Read Archive (SRA) under BioProject accession number PRJNA1515933 (BioSample accessions SAMN62592145–SAMN62592156; SRA run accessions SRR40278393–SRR40278401 and SRR40289325–SRR40289327). The metabolomics data generated in this study are available from the corresponding author upon reasonable request. All other data supporting the findings of this study are available within the article and its Supplementary Materials.

## Supplementary Materials

The following supporting information can be downloaded at: https://www.mdpi.com/article/10.3390/biology15171500/s1, Figure S1: Quality control of raw transcriptome sequencing data, Figure S2: GO enrichment analysis of differentially expressed genes across pairwise tissue comparisons, Figure S3: KEGG pathway enrichment of differentially accumulated metabolites, Table S1: Full list of differentially expressed genes identified in this study, Table S2: Full list of differentially accumulated metabolites identified in this study.
